# Enhancing Oil Content in Oilseed Crops: Genetic Insights, Molecular Mechanisms, and Breeding Approaches

**DOI:** 10.3390/ijms26157390

**Published:** 2025-07-31

**Authors:** Guizhen Gao, Lu Zhang, Panpan Tong, Guixin Yan, Xiaoming Wu

**Affiliations:** 1 Guangdong Provincial Key Laboratory of Conservation and Precision Utilization of Characteristic Agricultural Resources in Mountainous Areas, School of Life Science, Jiaying University, Meizhou 514015, China; gaoguiz0610@163.com (G.G.);; 2 Key Laboratory of Biology and Genetic Improvement of Oil Crops, Ministry of Agriculture and Rural Affairs, Oil Crops Research Institute of Chinese Academy of Agricultural Sciences, Wuhan 430062, China

**Keywords:** oilseed crops, oil content, genetic basis, molecular regulation, breeding

## Abstract

Vegetable oils are essential for human nutrition and industrial applications. With growing global demand, increasing oil content in oilseed crops has become a top priority. This review synthesizes recent progress in understanding the genetic, environmental, and molecular mechanisms regulating oil content, and presents biotechnological strategies to enhance oil accumulation in major oilseed crops. Oil biosynthesis is governed by intricate genetic–environmental interactions. Environmental factors and agronomic practices significantly impact oil accumulation dynamics. Quantitative trait loci (QTL) mapping and genome-wide association studies (GWAS) have identified key loci and candidate genes involved in lipid biosynthesis pathways. Transcription factors and epigenetic regulators further fine-tune oil accumulation. Biotechnological approaches, including marker-assisted selection (MAS) and CRISPR/Cas9-mediated genome editing, have successfully generated high-oil-content variants. Future research should integrate multi-omics data, leverage AI-based predictive breeding, and apply precision genome editing to optimize oil yield while maintaining seed quality. This review provides critical references for the genetic improvement and breeding of high- and ultra-high-oil-content varieties in oilseed crops.

## 1. Introduction

Vegetable oils are an excellent source of essential fatty acids, which are indispensable for human health. They are highly digestible, efficiently absorbed, and contain low cholesterol content. Moreover, vegetable oils are rich in a variety of essential nutrients, including proteins, vitamins, plant sterols, and polyphenols. These nutrients provide crucial energy and comprehensive nutritional support for maintaining human health [1]. Beyond their nutritional roles, vegetable oils have emerged as sustainable alternatives in various industrial applications, including biolubricants [2], hydrogel technology [3], UV-curing resins [4], and polyurethane adhesives [5]. Due to their dietary, nutritional, and industrial applications, vegetable oils have garnered substantial global interest. The major oilseed crops cultivated globally include soybean, palm, rapeseed, sunflower, peanut, cottonseed, sesame, linseed, castor, and safflower. Ensuring the sustainable production and utilization of these crops remains a key global challenge in addressing food security and nutritional needs.

**Figure 1:**
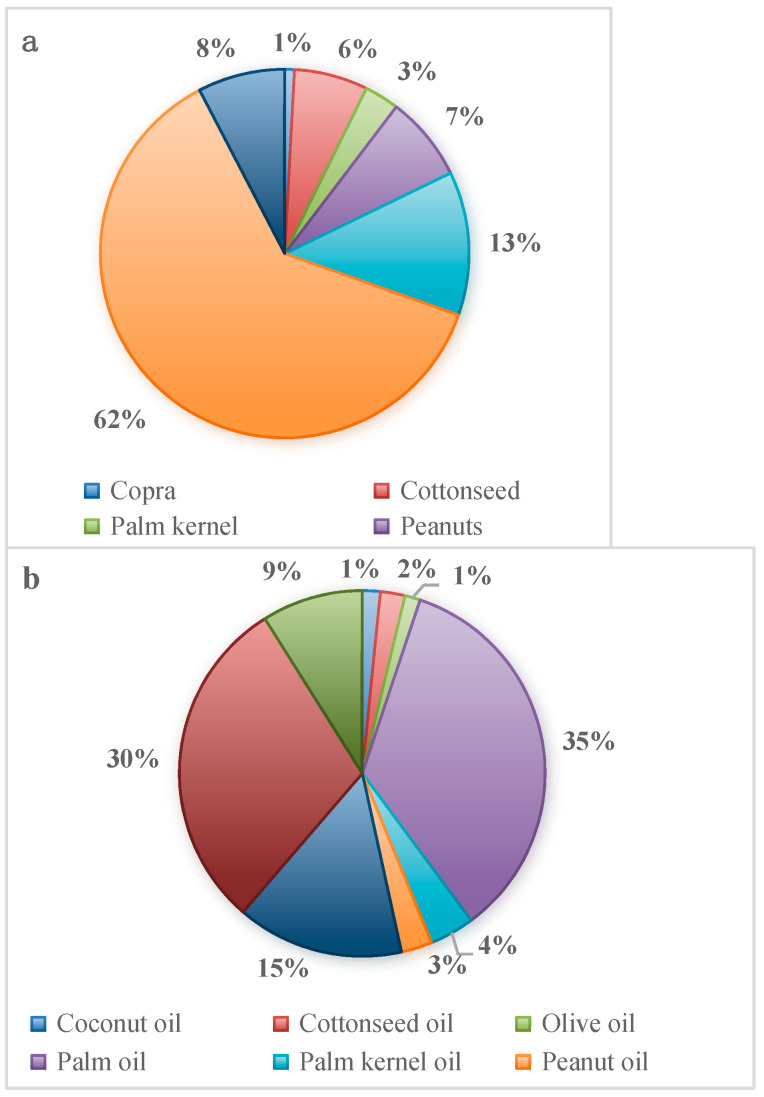
World oilseed (**a**) and vegetable oils (**b**) supply and distribution in 2024/2025.

In 2024/2025, the global oilseed supply reached 679.37 million metric tons (MMT). Soybeans dominated the market, contributing 420.76 MMT (approximately 62% of the total supply), followed by rapeseed (85.69 MMT, 13%), sunflower seed (52.02 MMT, 8%), peanut (50.60 MMT, 7%), and cottonseed (43.91 MMT, 6%). Correspondingly, the global supply of vegetable oils reached 228.98 MMT, with palm oil (35%), soybean oil (30%), rapeseed oil (15%), and sunflower seed oil (9%) constituting the largest share (Figure 1). (https://www.ers.usda.gov/data-products/oil-crops-yearbook, accessed on 24 March 2025).

With population growth and rising living standards, the demand for vegetable oil continues to escalate [6]. Oil extraction technology for high-yield oilseeds is critical to linking crop yields with market demand. Mainstream methods include traditional pressing and leaching, while innovative approaches like supercritical CO_2_ and ultrasonic-assisted extraction have improved efficiency and quality [7]. Despite technological advances, current systems still cannot meet rising global oilseed demand. The core constraint is the natural lipid synthesis capacity of oilseeds, limiting unit oil yield. Global oilseed consumption grows 2–3% annually, but limited arable land hinders expansion of planting areas, widening the supply–demand gap. Genetic modification to enhance oil content has become key to breaking this bottleneck, as it not only directly increases oil content but also improves crop stress resistance and adaptability [8]. In short, optimizing extraction technologies is a “symptomatic treatment” for boosting oil production, while genetic modification to enhance oil content is the “fundamental solution” to the supply–demand conflict.

The oil content generally ranges from 10% to 70% in oilseed crops [9,10] (Table 1). In general, a 1% increase in oil content can elevate overall yield by approximately 2.5% [11]. Given these pressing demands, developing novel high-oil cultivars is imperative. Previous studies have elucidated the genetic mechanisms and biosynthetic pathways governing oil content, alongside advancements in conventional and molecular-assisted breeding techniques. However, further investigation is essential to refine molecular strategies and accelerate the genetic improvement of oil content in oilseed crops.

## 2. Genetic and Environmental Regulation of Oil Content

### 2.1. Genetic Basis of Oil Content in Oilseed Crops

The genetic regulation of oil content in oilseed crops (e.g., soybean, rapeseed, sunflower, peanut and oil palm) is highly complex, governed by multiple genetic, environmental, and physiological factors. As a polygenic quantitative trait, oil content is influenced by embryonic, cytoplasmic, and maternal genetic components [12,13,14]. Broad-sense heritability (H^2^) estimates for oil content typically range from moderate to high (30–70%), indicating substantial genetic control [15]. However, narrow-sense heritability (h^2^) is often lower due to environmental modulation [16]. Notably, wild relatives and landraces frequently possess high-oil-content alleles that have been lost during domestication [17].

In rapeseed, oil accumulation is determined by the embryo genotype, cytoplasmic effects, maternal genotype, and genotype–environment interactions [18,19,20]. Additionally, photosynthetic efficiency and carbon flux from the silique wall during late seed development influence oil content [21]. Similarly, oil content is a quantitative trait regulated by multiple genes, with maternal additive and dominant effects combined in peanut [22,23] and maize [24]. Maternal inheritance also plays a key role in soybean [25], cotton [26] and sesame [27].

Overall, oil content in oilseed crops is a highly heritable yet intricate trait, governed by multigenic regulatory networks, epistatic interactions, and environmental interactions. Recent advances in genomics, gene editing, and precision breeding are accelerating the development of high-oil cultivars without compromising yield or quality. Furthermore, genotype–environment interactions remain a critical factor influencing oil accumulation. A two-year multi-location study of 52 peanut accessions revealed significant location effects on oil content. The average oil content was significantly higher in Florida (51.1%) and Georgia (50.7%) than in New Mexico (45.8%) [28]. Seed oil content (SOC) plasticity in *Brassica napus* was evaluated using 505 inbred lines across multi-environment trials over 4 years. The observed variation was primarily associated with three environmental factors: precipitation, diurnal temperature range, and ultraviolet B during the flowering or pod-filling stage [29].

### 2.2. Environmental Factors Influencing Oil Content in Oilseed Crops

The oil content of oilseed crops is determined by a dynamic interplay between genetic factors and environmental conditions. Key environmental variables include temperature, moisture, light intensity, soil fertility, and agronomic practices (Table 2) [30,31]. Significant variations in oil content have been observed for the same rapeseed cultivars grown across different locations and years, underscoring the strong environmental dependence of oil accumulation [32].

Temperature is the most critical environmental determinant of seed oil content. High temperatures accelerate seed maturation but may reduce oil content by prioritizing protein synthesis over lipid accumulation. They can also disrupt enzymes, altering fatty acid profiles. Low temperatures slow lipid biosynthesis but may increase unsaturated fatty acids to maintain membrane fluidity. Prolonged cold during seed filling reduces oil yield via slower metabolism [33]. For instance, elevated temperatures in Canada during 2012 reduced the average rapeseed oil content to 43.5%, compared to the 44.4% average recorded from 2007 to 2011 [34].

Light is another pivotal factor that significantly influences oil accumulation during seed maturation. Higher light enhances photosynthesis, providing more carbohydrates (sucrose) for lipid synthesis. Low light reduces sugar export to seeds, limiting oil accumulation [35]. Oil content increases by up to 4.7% when light intensity was elevated from 900 lx to 2880 lx during the 30th day of rapeseed flowering [36]. Conversely, peanut oil content decreased by 0.6–3.2% under shade treatment at the pod-filling stage [37]. Other environmental factors include altitude, soil fertility, planting density, sowing dates, cropping systems, and growth regulators [38,39,40]. Cultivation practices such as spring vs. summer sowing, film mulching vs. bare planting, crop rotation vs. continuous cropping, and drought stress vs. harvest times also play a role. For example, peanut oil content is higher during spring sowing, bare planting, continuous cropping, and moderate drought stress at the seedling stage [41].

Biotic and abiotic stresses, such as drought, saline-alkali stress, and pathogenic infections, impact oil accumulation by disrupting seed development [31]. Drought stress induces stomatal closure, reducing CO_2_ uptake and thereby limiting photosynthesis and carbon supply for oil synthesis. Waterlogging causes hypoxia, which inhibits root respiration, reducing ATP production and the availability of acetyl-CoA (a lipid precursor) [35]. For instance, prolonged rainfall during rapeseed flowering disrupts pollination, thereby reducing both seed yield and oil content. Ai et al. [42] reported that drought stress during the flowering and pegging stages of peanut significantly decreases oil content. In rapeseed, fungal diseases like Sclerotinia and downy mildew reduce seed yield, 1000-seed weight, and oil content [32].

Oil content of oilseed crops exhibits high sensitivity to environmental fluctuations. Maximizing oil yield requires optimal temperature regimes, balanced water management, sufficient solar radiation, and adequate soil nutrient availability. Breeding stress-tolerant cultivars and implementing precision agriculture practices represent viable strategies to mitigate the negative impacts of suboptimal environmental conditions.

**Table 2:** Effect of the main environmental factors on seed oil content.

Environmental Factor	Major Oilseeds	Impact on Oil Content	Reference
Temperature	Soybeans Rapeseed sunflower	±2–5%: -Heat stress lowers rapeseed oil content by 3–5%; -cold stress reduces soybean oil by 2–4%.	[34,43]
Water Availability	Sunflower peanuts sesame	±3–7%: -Drought reduces sunflower oil by 5–7%; -drought stress lowers peanut oil by 3–4%.	[42,44]
Soil Nutrients	Rapeseed Soybeans cottonseed	±2–4%: -High N lowers rapeseed oil by 2–3%; -optimal P increases soybean oil by 1–2%.	[45,46]
Light Intensity/Duration	Sunflower sesame canola	±3–6%: -Shading decreases sunflower oil by 4–6%; -long days increase canola oil by 2–3%.	[35]
Altitude	Rapeseed (high-altitude regions) peanuts	±4–8%: -Rapeseed oil content drops by 5–8% at >2000 m compared to lowlands.	[36]

## 3. Molecular Mechanisms of Oil Biosynthesis

### 3.1. High-Resolution QTL Mapping for Oil Content

Quantitative trait loci (QTL) mapping with linked markers provides a powerful approach for marker-assisted breeding of high oil content varieties. Multiple QTLs associated with oil content have been identified across major crops. In rapeseed, 173 QTLs for seed oil content have been mapped across 19 chromosomes, with notable clustering on chromosomes A02, A09, and C05 [47,48,49,50,51]. Due to the high environmental sensitivity of seed oil content, stable major-effect QTLs are challenging to isolate, necessitating multi-environmental phenotyping. Zhang et al. [52] analyzed oil contents across eight environments using a double–haploid population and identified 30 QTLs. Notably, the QTL qOC A10 was consistently detected in six environments, demonstrating its environmental stability. QTL analysis in a soybean recombinant inbred line (RIL) population identified five genomic regions associated with seed oil content distributed across five chromosomes. These regions collectively explained over 10% of the phenotypic variation over two consecutive years [53]. Zhang et al. [54] performed multi-environmental QTL mapping in 366 Chinese soybean germplasms, detecting 50, 98, and 50 QTLs for seed oil, oleic acid and linolenic acid contents, respectively, with 136, 283 and 154 associated alleles. In peanut, Liu et al. [55] constructed 186 recombinant inbred lines (RILs) from parents with divergent oil content and identified seven QTLs across four environments. Among these, qOCA08.1 was repeatedly detected and has been converted into a functional, molecular marker for high-oil peanut breeding. In maize, Yang et al. [56] constructed RILs genotyped with 228 molecular markers and identified 42 QTLs significantly associated with oil content and fatty acid composition. Five major QTLs were highlighted as potential targets for improving fatty acid composition and oil contents.

### 3.2. Genome-Wide Mapping of Oil Content Loci

The rapid advancement of high-throughput sequencing has enabled genome-wide association studies (GWAS) to become a powerful tool for identifying genes involved in lipid biosynthesis and regulation in oil crops. Integrative approaches combining GWAS, transcriptome-wide association studies (TWAS), genomic selection, and gene module analysis have significantly enhanced QTL detection. For example, Tang et al. [57] identified 27 QTLs significantly correlated with oil content in *Brassica napus* and functionally characterized a pair of homologous genes, *BnaPMT6*, which negatively regulate seed oil content. Hwang et al. [58] performed GWAS on 298 diverse varieties and detected 25 oil-associated SNPs, several of which overlapped with previously reported loci, demonstrating the high detection power of dense SNP marker systems. Similarly, Li et al. [59] analyzed 368 maize inbred lines using 1.03 million SNPs, uncovering 74 loci significantly associated with oil content and fatty acid composition. Remarkably, 26 loci collectively explained up to 83% of the phenotypic variation. In sesame, Wei et al. [27] constructed haplotype maps of 705 diverse varieties and performed GWAS on 56 agronomic traits, including oil content, nutritional quality, and yield. This study systematically identified six candidate genes involved in oil metabolism, including enzymes regulating lipid biosynthesis. The major and stable QTLs identified in these studies provide a foundation for fine mapping and candidate gene validation, offering promising targets for molecular breeding programs aimed at improving oil content in crops.

### 3.3. Key Enzymes and Pathways of Oil Biosynthesis

**Figure 2:**
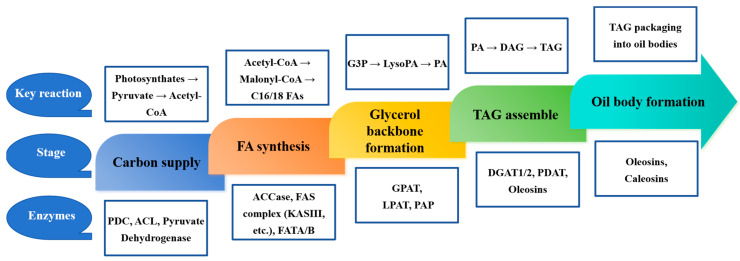
Key reaction and enzymes of oil biosynthesis in oilseed crops. ACCase: acetyl-CoA carboxylase. MCAT: fatty acid elongase. KAS: β-ketoacyl-ACP synthase. KAR: β-ketoacyl-ACP reductase. HAD: β-hydroxylacyl-ACP dehydrase. ENR: β-enoyl-ACP reductase. SAD: stearoyl-acyl carrier protein desaturase. FATA/FATB: fatty acid thioesterase. LACS: long-chain acyl-CoA synthase. LPCAT: lysophosphatidyl choline acyltransferase. FAD: fatty acid desaturase. GPAT: glycerol-3-phosphate acyltransferase. LPAAT: lysophosphatidic acid acyltransferase. PAP: phosphatidic acid phosphatase. DGAT: diacylglycerol acyltransferase.

The oil biosynthetic pathway has been extensively studied [60,61] and primarily consists of three key stages:

1. Fatty acid (FA) synthesis: this stage occurs in plastids, where acetyl-CoA carboxylase (ACCase) and fatty acid synthase (FAS) catalyze the conversion of acetyl-CoA into palmitic acid (16:0) and stearic acids (18:0).
2. Triacylglycerol (TAG) assembly via the Kennedy pathway: In the endoplasmic reticulum (ER), glycerol-3-phosphate is sequentially acylated by Glycerol-3-phosphate acyltransferase (GPAT), Lysophosphatidic acid acyltransferase (LPAT), and Diacylglycerol acyltransferase (DGAT, a critical rate-limiting enzyme) to form TAG. In oilseed, the Kennedy Pathway plays a pivotal role in oil biosynthesis by supplying phosphatidylcholine (PC), a key precursor for diacylglycerol (DAG), the direct substrate for TAG synthesis.
3. Phospholipid Editing: Phosphatidylcholine-derived acyl groups contribute to polyunsaturated fatty acids (PUFAs) through the action of PDAT (Phospholipid: diacylglycerol acyltransferase). (Figure 2, Table 3).

Oil synthesis pathways are highly conserved across species [30,62], yet divergent regulation mechanisms or key genes drive species-specific variations in oil content and composition [63,64]. For instance, overexpression of enzymes in the Kennedy Pathway has been shown to enhance TAG production [65].

### 3.4. Transcriptional Regulation of Oil Biosynthesis

Transcription factors (TFs) play pivotal roles in regulating seed oil accumulation, forming complex metabolic regulatory networks (Table 3) [66]. Key TFs involved in plant lipid synthesis include WRI1, LEC1, LEC2, FUS3, ABI3, DOF, and MYB [67]. Among these, WRI1 is the most extensively studied, with a relatively well-defined regulatory mechanism. In crops such as maize [68], rapeseed [69], and cotton [64], overexpression of the *WRI1* gene has been shown to significantly enhance seed oil content. LEC1 (LEAFY COTYLEDON1), a member of the NF-Y (Nuclear Factor Y) family, positively regulates oil accumulation. *LEC1* overexpression in rapeseed increases seed oil content by 7–16%, while inhibition of *LEC1* expression decreased oil content by approximately 10% [70]. Similarly, FUS3, LEC2, and ABI3 (plant-specific B3 family TFs) exhibit distinct regulatory patterns. In *B. napus,* mutations downstream of the *FUS3* B3 structural domain led to reduced oil content [71]. Conversely, *Arabidopsis thaliana* lines overexpressing *GmABI3* showed a 34.9% increase in TAG content compared to wild-type seeds [72].

**Table 3:** Key oil synthesis regulatory gene and transcription factor in oilseed crops.

Pathway	Gene	Gene Annotation	References
Fatty acid synthesis	ACCase	acetyl-CoA carboxylase	[73]
	MCAT	fatty acid elongase	[74]
	KAS	β-ketoacyl-ACP synthase	[70]
	KAR	β-ketoacyl-ACP reductase	[71,72]
	HAD	β-hydroxylacyl-ACP dehydrase	[72,75]
	ENR	β-enoyl-ACP reductase	[71,72]
	SAD	stearoyl-acyl carrier protein desaturase	[61,76]
	FATA FATB	fatty acid thioesterase	[60,77]
	LACS	long-chain acyl-CoA synthase	[61]
	LPCAT	lysophosphatidyl choline acyltransferase	[61]
	FAD	fatty acid desaturase	[60,61]
TAG synthesis	GPAT	glycerol-3-phosphate acyltransferase	[78]
	LPAAT	lysophosphatidic acid acyltransferase	[79]
	PAP	phosphatidic acid phosphatase	[80]
	DGAT	diacylglycerol acyltransferase	[55]
Transcription factors	WR11	AP2/EREBP transcription factor	[59,68,69]
	LEC	AP2/B3-like transcriptional factor	[71]
	ZF	Zinc finger transcription factor	[81]
	FUS3	FUSCA3	[71]
	ABI3	ABSCISIC ACID INSENSITIVE 3	[72]
	DOF	Dof zinc finger protein	[61]
	ZIP123	bZIP transcription factor	[82]
	MYB	MYB transcription factor	[81]

### 3.5. Post-Transcriptional and Epigenetic Regulation of Oil Biosynthesis

Post-transcriptional regulation refers to a series of molecular processes that modulate gene expression after transcription (i.e., from pre-mRNA processing to protein translation, modification, or degradation). In oilseed crops, these processes play a critical role in fine-tuning the complex network of oil biosynthesis, which involves carbon flux allocation, fatty acid synthesis, triacylglycerol assembly, and lipid storage. Post-transcriptional mechanisms interact dynamically to coordinate the activity of key enzymes and regulatory factors, ensuring efficient oil accumulation while responding to environmental cues. MicroRNAs (miRNAs) represent a key regulatory layer: miR156, miR172, and miR159 directly target transcription factors such as WRI1 and LEC2, both critical for fatty acid biosynthesis and TAG assembly [83,84]. For instance, miR396 suppresses lipid accumulation by downregulating *GL2*, a negative regulator of FA synthesis [83]. Conversely, transgenic *Arabidopsis* overexpressing miR319-resistant *TCP4 (rTCP4)* exhibited elevated TCP4 levels that downregulated *AtWRI1* target genes and promoted seed oil contents compared to wild-type plants [85]. Phosphoenolpyruvate carboxylase (PEPC) plays a pivotal role in linking carbon metabolism to lipid biosynthesis in oilseed crops by dynamically regulating carbon flux allocation and interacting with key enzymes and regulatory networks. PEPC catalyzes the irreversible carboxylation of phosphoenolpyruvate (PEP) to oxaloacetate (OAA), a critical step in shuttling carbon from glycolysis into lipid synthesis. This process occurs primarily in the cytosol and is essential for maintaining the acetyl-CoA pool required for fatty acid elongation and triacylglycerol assembly. RNA interference (RNAi)-mediated suppression of PEPC expression has been shown to enhance oil accumulation in cottonseeds [86]. In *B. juncea*, long non-coding RNAs (lncRNAs) act as miRNA sponges (e.g., sequestering miR858), thereby derepressing MYB transcription factors that activate lipid-related genes [84].

Tandem CCCH zinc finger proteins (e.g., soybean GmZF392) stabilize lipid biosynthesis mRNAs (e.g., DGAT1, PDAT1) by binding to their 3′ untranslated regions (UTRs). This mechanism enhances seed oil content by 10–15% in transgenic plants [87]. In *Perilla frutescens,* alternative splicing events regulate fatty acid desaturases (*FAD2* and *FAD3*), which in turn modulate α-linolenic acid (ALA) biosynthesis [88]. Under stress conditions, phosphorylation of ribosomal proteins and eukaryotic initiation factors (eIFs) fine-tunes the translation efficiency of lipid biosynthetic enzymes, directing cellular resources toward lipid storage pathways [89,90]. Collectively, these post-transcriptional regulatory mechanisms dynamically coordinate the expression of key enzymes, optimizing resource allocation for lipid accumulation, particularly under environmental stress.

Epigenetic modifications dynamically regulate chromatin accessibility and gene expression without altering DNA sequences. DNA methylation and histone modifications modulate chromatin accessibility, thereby influencing oil accumulation by silencing or activating lipid-related genes during seed development. For instance, hypomethylation at the promoters of *FAD2* and *FAE1* in *B. napus* correlates with increased polyunsaturated fatty acid (PUFA) synthesis under low-temperature stress [91]. A stochastically hypomethylated population of selfed lines (BraRoAZ) in *B. rapa*, comprising 1000 E2 sibling lines, was constructed using 5-azacytidine (5-AzaC). Forward screening for multiple agronomic traits revealed increased seed protein content and decreased oil content in this population [92]. In *Arabidopsis,* histone acetylation (e.g., H3K9ac) at the *WRI1* and *DGAT1* loci promotes transcriptional activation, whereas deacetylation mediated by HDACs suppresses lipid synthesis under nutrient-limited conditions [93,94]. Conversely, repressive histone marks (H3K27me3) silence *TT1* and *TT2*, which inhibit lipid droplet formation, thereby indirectly increasing oil content [83]. In sunflower, SWI/SNF complexes reposition nucleosomes at *HaWRI1* and *KASII* promoters, enabling transcriptional machinery access during peak oil accumulation [95].

## 4. Biotechnological Strategies for Elevating Oil Content

Enhancing oil accumulation and modulating fatty acid composition in oilseed crops represent central objectives of agricultural biotechnology. The Kennedy Pathway and associated lipid biosynthesis networks serve as prime targets for genetic engineering to improve both oil yield and quality. Recent biotechnological advancements have significantly advanced the enhancement of oil content in major oilseed crops, addressing global demands for food, biofuels, and industrial applications.

### 4.1. Breeding Strategies for Enhancing Oil Content

Advances in new sequencing technologies, along with the assembly and publication of reference genomes for oilseed crops (e.g., rapeseed, maize, soybean, and peanut), have spurred the transition from traditional hybrid breeding to precision molecular and design-based approaches (Table 4). For instance, Janila et al. [96] employed molecular marker-assisted backcrossing (MABC) and molecular marker-assisted selection (MAS) to develop peanut germplasm with divergent oil content, including 27 high-oil (53~58%) and 28 low-oil content (42~50%) accessions. Similarly, Zhao et al. [97] generated a stable ultra-high-oil soybean line (25.42%) by introducing the antisense *PEP* gene via *Agrobacterium*-mediated transformation. More recently, CRISPR/Cas9 technology has enabled precise modification of lipid metabolism genes in crops such as rapeseed, *Arabidopsis*, peanut, and soybean [98,99]. For example, knockout of *Bnlpat2* and *Bnlpat5* in rapeseed reduced oil content by 32% and 29%, respectively, while the double mutant (*Bnlpat2/Bnlpat5*) exhibited a 39% reduction [100].

### 4.2. Genetic Engineering for Enhancing Oil Content

In *Arabidopsis*, over 700 genes are involved in fatty acid metabolism [80]. Multiple key enzyme genes in the oil synthesis pathway have been successfully cloned and functionally characterized, including *ACCase, MCAT, KAS, KAR, HAD, SAD, FAD, GPAT, PDAT,* and *LPAAT* (Table 3). Heterologous expression of *ACCase* in *B. napus* increases oil content by ~6% [73]. MCAT mediates the acyl transfer step in fatty acid synthesis; overexpression in *Arabidopsis* enhanced both oil content and yield, whereas expression inhibition reduced oil content and caused growth retardation [74]. Similarly, overexpression of *AtGPAT7* and *AtGPAT9* in *Arabidopsis* increased seed oil content by 5–9% [78]. LPAAT, a rate-limiting enzyme in TAG biosynthesis, increases oil content by over 25% when its enzyme activity is enhanced in rapeseed [79]. PDAT facilitates the conversion of oleic acid into triacylglycerol; transferring the castor-specific gene *PDAT1-2* into *Arabidopsis* significantly promotes hydroxy fatty acid accumulation in seeds by up to 25% [101]. DGAT is the rate-limiting enzyme for the final step of TAG synthesis, and overexpression of *BnaDGAT* increases oil content by 4.24–5.80% in *Arabidopsis* [55].

**Table 4:** Strategies for improving oil content in oilseed crops.

Strategies	Elevation of Seed Oil Content	Advantages	Disadvantages	Reference
Conventional Breeding (e.g., screening, hybridization)	- Soybean from 18–22% to 22–25% -sesame from 50–55% to 52–57% - Safflower from 28–32% to 30–35% - peanut from 45–50% to 48–55%	- Low cost - Expands genetic variation; - Ensures adoption in local agro-ecosystems	- Slow progress (5–8 generations) - Limited genetic gain - Heterosis effects vary across environments	[102,103,104,105]
Mutagenesis Breeding	- Sunflower from 38–42% to 45–48% - Linseed: 3–6% increase (from 35–40% to 36–42%)	- Creates novel variation - No regulatory barriers; - Suitable for orphan crops	- Requires large mutant populations, labor-intensive screening - Random mutations may introduce undesirable traits	[106,107]
Marker-Assisted Selection (MAS)	- Rapeseed from 40–45% to 47–50% - Sunflower from 40–45% to 42–48%	- Faster than conventional - Targets specific loci - Low cost; - Applicable to non-transgenic varieties; - Maintains genetic diversity	- Requires well-characterized genetic maps - Marker-trait - Linkage may break	[108,109]
Genomic Selection (GS)	- Peanut from 45–48% to 50–53% - Cottonseed from 18–22% to 19–24%	- Utilizes whole-genome markers - Higher prediction accuracy - Accelerates breeding cycles (3–4 generations faster than MAS)	- Requires large training population - Computationally intensive - Less effective for traits with low heritability	[96,110]
Transgenic Approach	- Rapeseed: 8–12% increase (from 40–45% to 43–50%) - Palm: 5–8% increase (from 45–50% to 47–54%)	- Direct genetic improvement - Stable heritability	- Regulatory challenges - Public acceptance issues	[73,111]
Gene editing	- Soybean: 10–15% increase (from 18–22% to 20–25%) - Camelina from 35–38% to 42–45%	- Precise editing - No foreign DNA	- Off-target effects - Regulatory restrictions in many regions - High technical cost for multiplex editing	[112,113]

## 5. Future Prospects

### 5.1. Further Discovery and Exploitation of Functional Genes

Despite the identification of numerous QTL and molecular markers associated with oil content, their application in breeding and agriculture remains limited. Challenges such as variability in genotype frequency and germplasm diversity restrict the large-scale utility of certain genes or markers. Future research should prioritize the following directions:

Firstly, integration of genetic resources and genomic selection: establishing a genome-wide selection platform will facilitate the systematic identification and utilization of genes contributing to seed oil content. A critical first step involves expanding the collection and characterization of high-oil germplasm resources, as genetic diversity is fundamental for breeding advancements. Future research could employ multi-omics approaches, integrating transcriptomics, proteomics, and metabolomics data, to elucidate the regulatory networks underlying oil biosynthesis. Additionally, comparative genomics across different oil-producing species, like *Arabidopsis*, rapeseed, and palm, can uncover conserved genetic pathways and species-specific genes involved in oil production, offering new targets for genetic manipulation.

Secondly, functional validation of candidate genes: while GWAS has uncovered numerous loci linked to oil accumulation in oilseed crops, many lack candidate gene annotation or experimental validation. Future studies must focus on elucidating the biological roles of these loci through targeted gene identification and functional analyses.

### 5.2. Genome Editing as a Transformative Tool for Molecular Design Breeding

The CRISPR/Cas9 genome editing system has emerged as a transformative technology for crop improvement, offering unprecedented precision in genetic modification [114]. In rapeseed, CRISPR/Cas9-mediated editing has been successfully applied to modify key genes involved in fatty acid biosynthesis, fungicide resistance, and disease resistance, significantly advancing our understanding of the genetic basis underlying these agronomically important traits [115].

Despite these advances, the application of genome editing in oilseed crops faces several technical challenges. Current limitations include relatively low editing efficiency, transformation difficulties in most oilseed species, and the lack of efficient regeneration protocols for many cultivars. These constraints hinder the widespread implementation of genome editing in molecular breeding programs.

To overcome these barriers, future research should prioritize the development of genotype-independent transformation methods and the establishment of high-throughput screening protocols. Additionally, improving regeneration efficiency for edited cells will be crucial for accelerating the breeding process. Addressing these challenges will unlock the full potential of genome editing for precision breeding in oil crops, paving the way for the development of superior varieties with enhanced oil quality and yield.

### 5.3. AI-Driven Polymerization Breeding

Over the past two decades, a comprehensive database platform has been established, and several key regulatory genes related to oil content have been successfully cloned. However, it is difficult to effectively regulate oil content by simply changing the expression level of one or more genes, as oil content is controlled by multiple genes. Therefore, combining multi-omics analysis (genome, transcriptome, proteome, and metabolome) and transgenic technology is a future trend.

Machine learning (ML) algorithms are widely used to predict oil content in oilseed crops. In sunflower, oil yield prediction (OYP) via ML algorithms enables breeders to identify desirable new hybrids with high oil yield and their associated characteristics. For this purpose, a dataset comprising 1250 hybrids was used, with 70% randomly selected for model training and the remaining 30% for model testing and performance assessment. The results demonstrated that ML not only holds great potential for oil yield prediction but also facilitates the selection of parental lines for hybrid production [116]. The size of the training population is critical for achieving statistically robust predictions. Larger populations enhance model generalizability, particularly for complex traits like oil content. Small datasets may lead to overfitting, though this can be mitigated through cross-validation or synthetic data augmentation. In genomic selection, a reference population of 500+ genotypes is often recommended to ensure robust predictions [117].

ML algorithms leverage genomic datasets (SNPs, QTLs) associated with lipid biosynthesis genes to identify elite genotypes with superior oil content potential. By integrating multi-omics data (genomics, transcriptomics, metabolomics, and phenomics), artificial intelligence (AI) constructs predictive models that decipher the interplay between genetic regulators, metabolic networks, and environmental variables governing oil accumulation. This systems-level analysis enables the identification of key molecular drivers and environmental optima for maximizing oil biosynthesis in oilseed crops.

## Figures and Tables

**Table 1:** Oil content of different oilseed crops.

Crop	Oil Content Range (%)	Crop	Oil Content Range (%)
Soybean	18–28	Cottonseed	15–25
Palm kernel	45–55	Sesame	45–70
Rapeseed	40–65	Linseed	35–45
Sunflower	40–50	Castor	40–60
Peanut	45–62	Safflower	35–50

## Data Availability

No new data were created or analyzed in this study.
